# Supplementary material for: A Protein Microarray-Based Respiratory Viral Antigen Testing Platform for COVID-19 Surveillance
Source: Biomedicines. 2022 Sep 9;10(9):2238. doi: 10.3390/biomedicines10092238 (PMC9496200; doi:10.3390/biomedicines10092238)

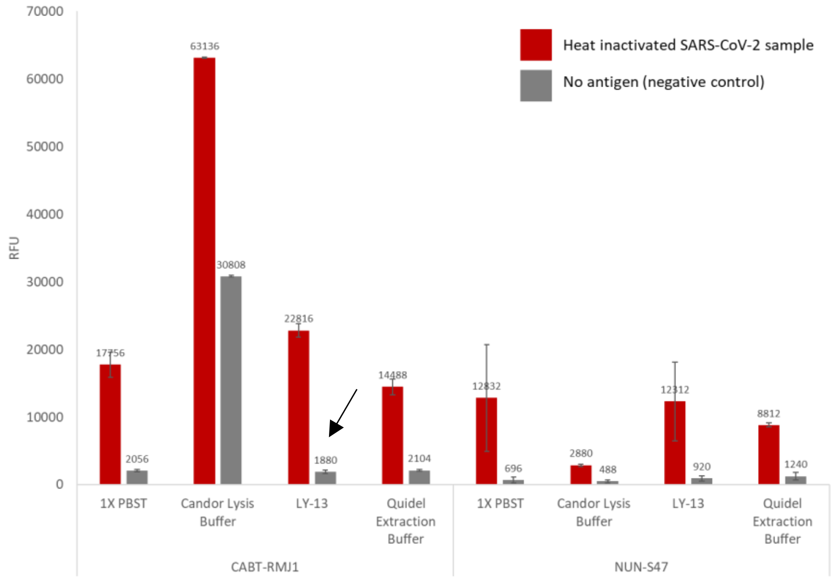

Figure S1. Test of lysis buffers for the inactivated SARS-CoV-2 sample. Using the same capture antibody from the pair number 1 from Table S2 (NUN-S46; reagent number **14 Table S1**), two detection antibodies (CABT-RMJ1 and NUN-S47; reagent number 13 and 15 **Table S1**) and four lysis buffers (1X PBST, Candor Lysis Buffer, LY-13, and Quidel Extraction Buffer) were tested against heat inactivated SARS-CoV-2 sample in all possible combinations. The combination of LY-13 and CABT-RMJ1 detection antibody resulted in the highest signal-to-noise ratio (SNR) (black arrow) in the CABT-RMJ1 subgroup with the least variation. A single experiment with two technical replicates were performed. Error bars represent standard deviation from the technical replicates.

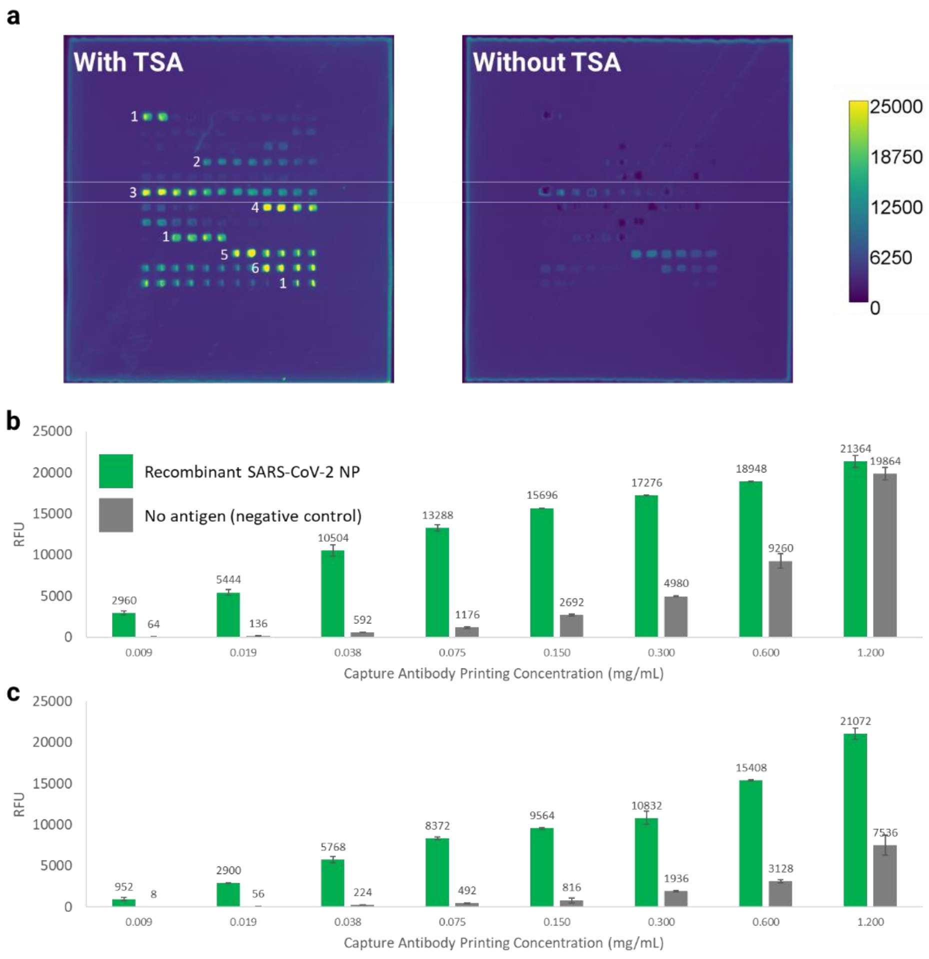

Figure S2. TSA on the protein microarrays. Streptavidin-HRP conjugates were used for TSA. The detection scheme to capture SARS-CoV-2 NP using TSA is shown in **Figure 1d**. TSA was performed to compare the fluorescence signals to those from streptavidin-fluorophore approach, shown in **Figure 1c**. The same concentration of the recombinant SARS-CoV-2 NP was tested for TSA and streptavidin-fluorophore approach. (**a**) Representative pad images between the TSA with Alexa Fluor 488 tyramide reagents treated pad (left) and the streptavidin-Alexa Fluor 488 treated pad (right) are shown. The two white lines indicate the location of the optimal anti-SARS-CoV-2 NP capture antibody (label 3) at different printing concentrations. Each label indicates the following reagents: label 1- fiducials, label 2, 3, 5, and 6 - anti-SARS-CoV-2 NP capture antibodies (reagent number 8, 14, 11, and 12 in **Table S1**, respectively), and label 4 - recombinant SARS-CoV-2 NP. A single experiment with two different tyramide reagent incubation periods were tested. (**b**) 10-minute incubation and (**c**) 2-minute incubation are shown. Two technical replicates were performed for each condition. The 2-minute tyramide reagent incubation resulted in the higher SNR ratio than the 10-minute incubation.

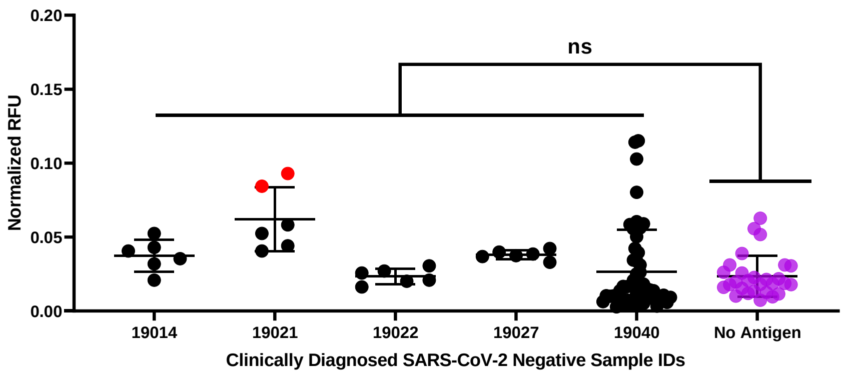

Figure S3. Negative SARS-CoV-2 clinical sample controls. A total of 5 negative clinical samples were tested on the protein microarrays. Normalized RFUs from each negative clinical sample were compared to the background (No Antigen) control. One-

way ANOVA, followed by Tukey's multiple comparison test, was performed for statistical analysis. The two data points (red circles) from the sample ID 19021 were excluded from the statistical analysis due to the high fluorescence background on the nitrocellulose pad. No statistical significance was observed between the negative clinical sample and the background (No Antigen) control. The sample ID 19040 was mainly used to calculate the thresholds to test RT-PCR-confirmed positive SARS-CoV-2 clinical samples on the protein microarray. For the sample ID 19014, 19021, 19022, and 19027, a single experiment with six technical replicates were performed. For the sample ID 19040 and the No Antigen control, at least three independent experiments were performed. p-values between the No Antigen control and the sample ID 19014, 19021, 19022, 19027, and 19040 are 0.7303, 0.2727, > 0.9999, 0.6912, and 0.9916, respectively.

Table S1. A list of antibodies and recombinant proteins used in this study for antigen detection.

Reagent Number	Product Name	Antibody / Antigen	Antibody Purpose	Catalog Number	Vendor
1	SARS-CoV-2 (2019-nCoV) Nucleocapsid-His recombinant Protein	Antigen	NA	40588-V08B	Sino Biological
2	SARS-CoV-2 (2019-nCoV) Spike RBD-His Recombinant Protein	Antigen	NA	40592-V08B	Sino Biological
3	Influenza A H1N1 (A/California/04/2009) Hemagglutinin / HA Protein (His Tag)	Antigen	NA	11055-V08H2	Sino Biological
4	Influenza B (B/Florida/4/2006) Hemagglutinin / HA Protein (His Tag)	Antigen	NA	11053-V08H	Sino Biological
5	Human coronavirus (HCoV-OC43) Nucleocapsid Protein (His Tag)	Antigen	NA	40643-V07E	Sino Biological
6	SARS-CoV-2 (2019-nCoV) Spike Neutralizing Antibody, Mouse Mab	Antibody	Capture Antibody	40591-MM43	Sino Biological
7	SARS-CoV/SARS-CoV-2 Spike antibody, Chimeric Mab	Antibody	Detection Antibody	40150-D004	Sino Biological
8	SARS-CoV/SARS-CoV-2 Nucleocapsid Antibody, Mouse Mab	Antibody	Capture Antibody	40143-MM05	Sino Biological
9	SARS-CoV/SARS-CoV-2 Nucleocapsid Antibody, Mouse Mab	Antibody	Capture Antibody	40143-MM08	Sino Biological
10	SARS-CoV/SARS-CoV-2 Nucleocapsid Antibody, Rabbit Mab	Antibody	Detection Antibody	40143-R001	Sino Biological
11	Anti-SARS-CoV-2 Nucleoprotein monoclonal antibody	Antibody	Capture Antibody	CABT-MMB1	Creative Diagnostics
12	Recombinant Anti-SARS-CoV-2 NP monoclonal antibody	Antibody	Capture Antibody	CABT-MMB2	Creative Diagnostics
13	Anti-SARS-CoV-2 Nucleoprotein monoclonal antibody	Antibody	Detection Antibody	CABT-RMJ1	Creative Diagnostics
14	Anti-SARS-CoV-2 Nucleocapsid Antibody, Mouse IgG1 (AS46) (Trehalose free)	Antibody	Capture Antibody	NUN-S46	ACRO Biosystems
15	Anti-SARS-CoV-2 Nucleocapsid Antibody, Mouse IgG1 (AS47) (Trehalose free)	Antibody	Detection Antibody	NUN-S47	ACRO Biosystems
16	Influenza A Virus Hemagglutinin / HA Antibody, Rabbit MA	Antibody	Capture Antibody	86001-RM01	Sino Biological
17	Influenza B Hemagglutinin / HA Antibody, Mouse Mab	Antibody	Capture Antibody	11053-MM09	Sino Biological
18	Human coronavirus (HCoV-OC43) Nucleocapsid Antibody, Rabbit PAb, Antigen Affinity Purified	Antibody	Capture Antibody	40643-T62	Sino Biological
19	Biotin-SP (long spacer) AffiniPure Goat Anti-Human IgG, Fcγ fragment specific	Antibody	Secondary Antibody	109-065-098	Jackson ImmunoResearch
20	Biotin-SP (long spacer) AffiniPure Goat Anti-Rabbit IgG, Fc fragment specific	Antibody	Secondary Antibody	111-065-046	Jackson ImmunoResearch
21	ChromPure Mouse IgG, whole molecule	Antibody	Control Antibody	015-000-003	Jackson ImmunoResearch
22	Human IgG Isotype Control	Antibody	Control Antibody	12000C	Thermo Fisher Scientific

NA: not applicable.

Table S2. Summary of SARS-CoV-2 antigen capture and detection antibody pairs to develop sandwich immunoassay on the protein microarray.

Pair	Capture Antibody Vendor	Capture Antibody Catalog Number	Capture Antibody Printing Concentration (mg/mL)	Antigen Target
1	Acro Biosystems	NUN-S46	1.200	SARS-CoV-2 NP
2	Creative Diagnostics	CABT-MMB1	0.600	SARS-CoV-2 NP
3	Creative Diagnostics	CABT-MMB2	1.200	SARS-CoV-2 NP
4	Sino Biological	40143-MM05	1.690	SARS-CoV-2 NP
5	Sino Biological	40143-MM08	1.735	SARS-CoV-2 NP
6	Sino Biological	40591-MM43	1.019	SARS-CoV-2 Spike RBD

Pair	Detection Antibody Vendor	Detection Antibody Catalog Number	Detection Antibody Probing Concentration (mg/mL)	Secondary Antibody
1	Acro Biosystems	NUN-S47	0.003	NA
2	Acro Biosystems	NUN-S47	0.003	NA
3	Acro Biosystems	NUN-S47	0.003	NA
4	Sino Biological	40143-R001	0.005	Biotinylated anti-rabbit antibody
5	Sino Biological	40143-R001	0.005	Biotinylated anti-rabbit antibody
6	Sino Biological	40150-D004	0.008	Biotinylated anti-human antibody

Pair	Detection Scheme	Total Incubation Time (hr)	Bmax	K_D (M)
1	Figure 1C	2.5	59496	7.327E-10
2	Figure 1C	2.5	52124	1.154E-09
3	Figure 1C	2.5	51497	2.693E-09
4	Figure 1B	3.5	60005	2.454E-09
5	Figure 1B	3.5	47574	2.737E-08
6	Figure 1B	3.5	45567	6.463E-12

NA: not applicable
RBD: receptor binding domain

The protein microarray chips tested the pairs 4, 5, and 6 were imaged using Perkin Elmer's ScanArray Express HT scanner.

Table S3. Summary of clinically diagnosed RT-PCR positive samples.

Patient ID	Sex	Age	Group	UTM	RT-qPCR Kits	Ct
18934	M	50Y	Group 1	Remel	Alinity m SARS-CoV-2 assay / Abbott RealTime SARS-CoV-2 assay	5.48
18812	M	84Y	Group 1	Hardy	Alinity m SARS-CoV-2 assay / Abbott RealTime SARS-CoV-2 assay	9.12
18880	F	63Y	Group 1	Remel	Alinity m SARS-CoV-2 assay / Abbott RealTime SARS-CoV-2 assay	14.23
18827	F	62Y	Group 1	Remel	Alinity m SARS-CoV-2 assay / Abbott RealTime SARS-CoV-2 assay	15.67
18826	F	21Y	Group 1	Remel	Alinity m SARS-CoV-2 assay / Abbott RealTime SARS-CoV-2 assay	17.66
18930	M	24Y	Group 1	Remel	Alinity m SARS-CoV-2 assay / Abbott RealTime SARS-CoV-2 assay	23.84
19071	F	56Y	Group 1	Remel	Alinity m SARS-CoV-2 assay / Abbott RealTime SARS-CoV-2 assay	29.91
19072	F	54Y	Group 1	Remel	Alinity m SARS-CoV-2 assay / Abbott RealTime SARS-CoV-2 assay	31.82
18958	M	16Y	Group 1	Remel	Alinity m SARS-CoV-2 assay / Abbott RealTime SARS-CoV-2 assay	33.70
18862	F	49Y	Group 2	Hardy	Simplexa COVID-19 Direct assay / ABI 7500 Fast Dx SARS-CoV-2 assay	12.20
18833	F	33Y	Group 2	Remel	Simplexa COVID-19 Direct assay / ABI 7500 Fast Dx SARS-CoV-2 assay	15.10
18911	M	81Y	Group 2	Remel	Simplexa COVID-19 Direct assay / ABI 7500 Fast Dx SARS-CoV-2 assay	14.40
18879	M	23Y	Group 2	Remel	Simplexa COVID-19 Direct assay / ABI 7500 Fast Dx SARS-CoV-2 assay	15.90
18829	F	48Y	Group 2	Remel	Simplexa COVID-19 Direct assay / ABI 7500 Fast Dx SARS-CoV-2 assay	19.40
18913	F	37Y	Group 2	Remel	Simplexa COVID-19 Direct assay / ABI 7500 Fast Dx SARS-CoV-2 assay	19.90
18959	M	45Y	Group 2	Remel	Simplexa COVID-19 Direct assay / ABI 7500 Fast Dx SARS-CoV-2 assay	31.50
18811	F	33Y	Group 3	Hardy	Xpert Xpress SARS-CoV-2 test / Xpert Xpress CoV-2/Flu/RSV plus	11.70
18817	F	20Y	Group 3	Hardy	Xpert Xpress SARS-CoV-2 test / Xpert Xpress CoV-2/Flu/RSV plus	15.90
18834	F	50Y	Group 3	Remel	Xpert Xpress SARS-CoV-2 test / Xpert Xpress CoV-2/Flu/RSV plus	20.10
18954	M	71Y	Group 3	Remel	Xpert Xpress SARS-CoV-2 test / Xpert Xpress CoV-2/Flu/RSV plus	26.30
18943	M	52Y	Group 3	Remel	Xpert Xpress SARS-CoV-2 test / Xpert Xpress CoV-2/Flu/RSV plus	27.60
18960	F	49Y	Group 3	Remel	Xpert Xpress SARS-CoV-2 test / Xpert Xpress CoV-2/Flu/RSV plus	35.40

Group 2 assays were tested on Thermo Fisher Applied Biosystems.
Each clinical sample was tested by one of the two assays indicated in the RT-qPCR Kits column.

Table S4. Microarray assay precision against heat inactivated SARS-CoV-2 sample

Concentration ($TCID_{50}$/mL)	Technical Replicates (n)	Average RFU	Standard Deviation	CV%
1.47E+02	2	976	91	9.27
2.81E+02	2	584	34	5.81
5.62E+02	2	836	6	0.68
7.36E+02	2	768	181	23.57
1.12E+03	2	524	28	5.40
2.25E+03	2	752	23	3.01
3.68E+03	2	1088	272	24.96
4.49E+03	2	744	11	1.52
8.98E+03	2	1416	147	10.39
1.80E+04	2	76	107	141.42
1.84E+04	2	1048	215	20.51
3.59E+04	2	1248	385	30.82
7.19E+04	2	1236	85	6.87
9.20E+04	2	1440	520	36.14
1.44E+05	2	2208	45	2.05
2.88E+05	2	2720	45	1.66
4.60E+05	2	14608	611	4.18
5.75E+05	2	6236	730	11.70
1.15E+06	2	19824	226	1.14
2.30E+06	4	53014	12446	23.48

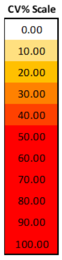

Supplement: Supplementary file 1 [file biomedicines-10-02238-s001.zip › biomedicines-1869440-supplementary.pdf]
